# Molecular epidemiology of methicillin resistant *Staphylococcus* species in healthcare workers of a blood bank in the Brazilian Amazon

**DOI:** 10.1186/s12866-021-02365-1

**Published:** 2021-11-04

**Authors:** Cristina Motta Ferreira, Roberto Alexandre Alves Barbosa Filho, Guilherme Motta Antunes Ferreira, Marcus Vinicius Guimarães de Lacerda, Cintia Mara Costa de Oliveira, Vanderson de Souza Sampaio, Lucyane Mendes Silva, Andreza Gomes Pascoal, William Antunes Ferreira

**Affiliations:** 1 Fundação Hospitalar de Hematologia e Hemoterapia do Amazonas – HEMOAM, Manaus, Brazil; 2 Universidade Federal do Amazonas – UFAM, Manaus, Brazil; 3 Programa de Pós-Graduação em Hematologia, Universidade do Estado do Amazonas - UEA, Manaus, Brazil; 4 Fundação de Medicina Tropical Dr. Heitor Vieira Dourado - FMT-HVD, Manaus, Brazil; 5 Instituto Leônidas e Maria Deane-Fiocruz, Manaus, Brazil; 6 Universidade do Estado do Amazonas - Programa de Pós-Graduação em Medicina Tropical, Manaus, Brazil; 7 Fundação de Vigilância em Saúde do Amazonas, Manaus, Brazil; 8 Fundação de Dermatologia Tropical e Venereologia Alfredo da Matta – FUAM, Manaus, Brazil

**Keywords:** *S. epidermidis*, Multirresistance, Oxacillin, ST, Clone

## Abstract

**Background:**

Healthcare workers are susceptible to colonization by multiresistant bacteria, which can increase the risk of outbreaks.

**Methods:**

Samples were collected from the nasopharynx, hands, and lab coats of healthcare workers. The phenotypic identification was carried out using a VITEK®2 rapid test system. PCR tests for the *mec*A gene and the sequencing of the amplicons were performed. *Staphylococcus epidermidis* and *Staphylococcus aureus* phylogenies were reconstructed using the Bayesian inference.

**Results:**

A total of 225 healthcare workers participated in this study. Of these, 21.3% were male and 78.7% female. *S. epidermidis* and *S.aureus* showed high levels of resistance to penicillin, ampicillin, erythromycin, tetracycline and cefoxitin. The prevalence of methicillin resistant *S. aureus* was 3.16% and methicillin resistant *S. epidermidis* was 100%. Multilocus sequence typing identified 23 new *S. epidermidis* sequence types, and one new allele and sequence type for *S. aureus*. The frequency of methicillin-resistant *S. epidermidis* in nursing and hemotherapy technicians as a percentage of the total number of healthcare workers was 5.8–3.1%, while the frequency of methicillin resistant *S. aureus* in hemotherapy technicians and biomedics, as a percentage of the total number of healthcare workers was 4.2–8.9%%.

**Conclusions:**

The healthcare workers at the city’s blood bank, even when taking the necessary care with their hands, body and clothes, harbour methicillin-resistant *S. aureus* and *S. epidermidis* sequence types, which, as a potential source of multidrug resistant bacteria, can contribute to nosocomial infections among hematological patients.

**Supplementary Information:**

The online version contains supplementary material available at 10.1186/s12866-021-02365-1.

## Background

Nosocomial infections continue to be a major clinical concern worldwide. In emerging countries, 1 in 100 hospitalized patients acquire such infections. Studies conducted in high-income countries identified that 5–15% of the hospitalized patients acquire healthcare-associated infections, which can affect from 9 to 37% of those admitted to intensive care [1]. In the USA, the Center for Disease Control and Prevention-CDC calculated that annually there were approximately 1.7 million hospital infections from all types of microorganisms resulting in 99,000 deaths [2].

Nosocomial infections can damage not only the health of patients but also the health of staff. Hospitalized patients, especially those in the intensive care unit (ICU), [3–6] are particularly susceptible to nosocomial infections due to their immune systems being compromised by indwelling devices, underlying diseases, and prolonged overuse of broad-spectrum antibiotics [7].

When there is a lack of adherence to standard precautions, healthcare workers, especially nursing professionals (who were associated in the majority of the cases) are certainly more susceptible to colonization by multi-resistant bacteria, and as a result may cause outbreaks [6, 8]. However, the most common form of transmission, which favors the dissemination of these bacteria among patients and healthcare workers, is through contact that involves the hands [6, 9, 10].

Staphylococci are the bacteria that are most frequently involved in hospital infections and colonize skin and nasal mucosa as their normal flora. Nasal carriage is one of the main sources for nosocomial infections among hospital staff [9, 11]. When colonization occurs by multi-resistant isolates, it becomes a risk factor for both the person who has been colonized and their contacts [10], and it is therefore of great clinical importance to identify which professionals are harbouring clones of dangerous, multi-resistant bacteria that are sources of nosocomial pathogenic clones [12]. Aproximately 30–94% of healthcare workers carry methicillin-resistant *Staphylococcus epidermidis* (MRSE) and 16.8–56.1% carry methicillin-resistant *Staphylococcus aureus* (MRSA) in their nasal mucosa [9, 13], indicating that they may serve as important reservoirs and potential spreaders of these bacteria to uncolonized susceptible patients as well as being a source of nosocomial infections [13–15]. In the city of Manaus, Amazonas state, Brazil, as yet, no data has been produced regarding prevalence and/or levels of antimicrobial resistance to Gram-positive bacteria sequence types (STs) that colonize healthcare professionals, hence the importance of this study. The only data is from a study performed by Ferreira et al. [16], which identified new multi-resistant clones of *S. epidermidis* ST365 that were isolated from immunosuppressed patients who had been admitted to the city’s blood bank, where this study was realized. Therefore, the aim of this study is the detection of multi-resistant *S.epidermidis* and *S. aureus*, their prevalence and phylogenetic relationships, and the results of antimicrobial susceptibility tests of a new sequence typing (ST) that were performed on healthcare workers at the Hematology and Hemotherapy Foundation of the Amazonas - HEMOAM.

## Methods

### Study population and collection of samples

This study was conducted at the city’s public hematology center in Manaus, which is responsible for the diagnosis and treatment of outpatients and inpatients with malignant and benign hematological diseases. The public hematology center is also responsible for the care and control of blood and blood derivatives that are made available for the residents of the city of Manaus.

The recruitment of the study subjects and sample collections were carried out during the subjects’ routine activities at HEMOAM, in 2015. Among the 400 healthcare workers at HEMOAM, 230 who maintained clinical and non-clinical contact with the patients agreed to participate in the study and signed the informed consent form. However, five healthcare workers were excluded due to the infeasibility of the samples during the development of the methodologies; therefore, 225 out of 400 subjects effectively participated in the study.

Three samples were taken from the nasopharynx, hands and lab coat of the subjects using three different sterile swabs that were placed in Stuart transport media (Labor Import, Guangzhou, China), and then transported to the Clinical Bacteriology Laboratory at HEMOAM.

### Cultivation, identification and susceptibility testing

The primary sowing was carried out with 5% sheep blood agar and mannitol salt agar (Himedia-Hexasystems, Mumbai, India), which was then incubated for 24 h at 35.4 °C. Single isolated colonies were selected and phenotypic identification of MRSA and MRSE was performed using a rapid test system (VITEK®2, bioMérieux, France).

An in vitro antibiotic susceptibility test was performed using the standardized Kirby–Bauer disc diffusion method on a Mueller-Hinton agar plate with Gram-positive multi-discs (Laborclin, Paraná, Brazil) containing penicillin (10 μg), gentamicin (10 μg), oxacillin (30 μg), sulfamethoxazol-trimethropim (25 μg), chloramphenicol (30 μg), ciprofloxacin (5 μg), clindamycin (2 μg), erythromycin (15 μg), rifampin (5 μg) and tetracycline (30 μg). The plates were incubated for 24 h at 35.4 °C (95.7 °F). Methicillin and vancomycin resistance was tested using Mueller-Hinton agar with E test (MIC) and streaks of oxacillin and vancomycin antibiotic at a concentration ranging from 0.016 to 256 μg/mL. Diameters of the zone of inhibition were measured and MIC interpretation of the reference values for sensitivity, reduced sensitivity or resistance to antibiotics were set according to the Clinical & Laboratory Standards Institute manual (CLSI, 2015) [17].

*Staphylococcus aureus* (*S. aureus*) ATCC 25923 was used for the susceptibility tests. Aliquots of the MRSA and MRSE isolates were stored at − 80 °C in a cryotube with Brain Heart Infusion (BHI) broth (Himedia, Hexasystens-Mumbai, India) + 20% glycerol, for further molecular testing.

### Molecular characterization

Methicillin resistant *S. aureus* and *S. epidermidis* isolates were submitted to molecular analysis and genomic DNA extraction was performed using the PureLink genomic DNA mini Kit (Invitrogen, CA, USA), and polymerase chain reaction was used to amplify the *mec*A gene, according to Jonas et al. [18]. Seven multilocus sequence typing (MLST) target genes were amplified using PCR according to the methods published for *S. epidermidis* and *S. aureus* available on the mlst.net webpage. The amplicons were sequenced with a 3130 Genetic Analyser (ABI 3130, Applied Biosystems, USA). The sequences were analysed using the Geneious software version 10.0.10 (Biomatters, New Zealand). Alleles and STs (sequencing types) were determined using the *S. epidermidis* (https://pubmlst.org/sepidermidis/) and *S. aureus* (http://saureus.beta.mlst.net/) MLST databases.

### Allelic diversity of ST

For the phylogenetic analysis of *S. aureus* and *S. epidermidis*, two local databases were built from all sequences annotated for each gene of the MLSTs (available in https://pubmlst.org), totaling 28,194 isolates for *S. aureus* and 1731 for *S. epidermidis*.

The sequences of the MLST genes were clustered and aligned with the *S. aureus* and *S. epidermidis* sequences isolated in this study (9 and 43 samples respectively), totaling 28,203 MRSA and 1774 MRSE samples, using the MAFFT tool (available in https://mafft.cbrc.jp/alignment/server) to the alignment. After the alignment of the sequences of each MLST gene, the Tajima neutrality test was performed in order to check whether the alleles were evolving in a stochastic manner or undergoing some form of selection [19]. The Tajima statistical test was performed with 1000 replicates, using the DnaSP 6 program, from the Universitat de Barcelona, current beta version: 6.12.03 (February 26, 2019), available at http://www.ub.edu/dnasp/.

### Phylogenetic analysis

We used Markovian Chain Sampling (considering 1 × 10^6^ generations and the evolutionary evolutive model GTR) [20] from the Mr. Bayes software version 3.2.6 [21] to estimate the phylogenetic relationships between the samples from the alignment of the concatenated MLST gene sequences isolated in this study. The phylogenetic relationships of the genetic distances between the STs were estimated using the comparative eBURST V3 software, which employed the eBURST algorithm (http://www.phyloviz.net/goeburst).

## Results

Of the 400 healthcare workers at HEMOAM, 225 (56.2%) were included in the study, of which 48 (21.3%) were male and 177 (78.7%) female. The mean age was 44 years of age. Of the total, 114 healthcare workers had regular contact with hospitalized patients or attended patients at the HEMOAM outpatient clinic. The distribution of the healthcare workers by type of work performed is shown in Table 1. Nursing technicians (*P*-value = 0.001) and administrative workers (*P*-value = 0.037) were associated with MRSE carriage, while for MRSA, the greatest association was with administrators (*P*-value = 0.036).

**Table 1:** Distribution of the healthcare workers by profession and presence of MRSA/MRSE

Profession	n	%	MRSA			MRSE		
			n	%	***P***-value	n	%	***P***-value
Physician	13	5.8	0	0	0.616	2^a^	4.4	0.291
Biochemist	16	6.3	0	0	0.549	1	2.2	0.184
Nurse	15	6.8	1	11.1	0.429	2	4.4	0.481
Nursing technician	21	9.3	0	0	0.451	12^a^	26.8	**0.001**
Nursing assistant	2	0.9	0	0	0.930	1	2.2	0.325
Lab technician	24	10.8	1	11.1	0.601	4	8.9	0.571
Hemotherapy technician	53	23.6	5^a^	55.6	0.091	^a^	15.6	0.112
Administrative staff	48	21.3	0	0	0.142	4	8.9	**0.037**
Social worker	5	2.3	0	0	0.833	3^a^	6.7	0.217
Biomedic	5	2.3	1	11.1	0.167	1	2.2	0.628
Ambulance driver	4	1.8	0	0	0.864	1	2.2	0.546
Psychologist	3	1.4	0	0	0.897	1	2.2	0.446
Physiotherapist	3	1.3	0	0	0.897	1	2.2	0.446
Cleaning staff	3	1.3	0	0	0.897	3^a^	6.7	0.082
Librarian	2	0.9	0	0	0.930	0	2.2	0.675
Lawyer	2	0.9	0	0	0.930	0	0	0.675
Pharmacist	1	0.5	0	0	0.964	0	0	0.822
Dentist	1	0.5	0	0	0.964	0	0	0.822
Biologist	1	0.5	0	0	0.964	1	2.2	0.178
Chemist	1	0.5	0	0	0.964	0	0	0.822
Administrator	1	0.5	1	11.1	**0.036**	0	0	0.822
Statistician	1	0.5	0	0	0.964	1	2.2	0.178
**Total**	**225**	**100**	**8**	**100**	**–**	**40**	**–**	**–**

^a^These participants had two positive samples

The most frequent staphylococci species isolated were *S. epidermidis* (*n* = 45); *S. aureus* (*n* = 284), and other saprophytic isolates (*n* = 319) were classified *as Staphylococcus* spp. The prevalence of MRSA was 3.1% (9/284), MRSE was 100% (45/45), and MSSA was 96.8% (275/284).

An antimicrobial susceptibility test (AST) for MRSE (*n* = 45) showed that 97.7% (44/45) were resistant to penicillin, 37.8% (17/45) to tetracycline, 35.5% (16/45) to sulfazotrim, 62.3% (28/45) to erythromycin, and 100% to oxacillin and cefoxitin (MRSE) with a minimum inhibitory concentration value (MIC E test) ranging from 0.25 μg/mL to over 256 μg/mL. No association was identified between MRSE and gender, age, or profession (Supplementary material - S1).

The AST for MRSA (*n* = 9) showed that 88% were resistant to ampicillin, 67% (6/9) to tetracycline, 44% (4/9) to erythromycin and 100% to penicillin, oxacillin and cefoxitin, with a MIC E test ranging from 24 μg/mL to over 256 μg/mL. Resistance to vancomycin was not detected, and phenotypic resistance to methicillin was confirmed by the detection of the *mec*A gene.

Multilocus sequence typing performed on 45 isolates of the *S. epidermidis* detected 22 new STs: ST661–669, ST643–653, ST670, and ST671. The remaining STs (*n* = 23) that were identified were ST20 (*n* = 6), ST296, ST61, ST152, ST173 (*n* = 2), ST194, ST17 (*n* = 3), ST2, ST153, ST255, ST185, ST69, ST59 (*n* = 2) and ST642.

Multilocus sequence typing performed for *S. aureus* detected one new gene (allele *pta* 680) and a new ST 5800 (ID 34725). The remaining STs (*n* = 8) were ST5 (*n* = 3), ST15 (*n* = 2), ST30, ST1993 and ST4952 (GenBank accession numbers: MW115983–115989; 115,990–115,996; 115,997–116,003; 116,004–116,010; 116,011–116,017; 116,018–116,024; 116,025–116,031; 116,032–116,038; 116,039–116,045).

The distribution of the ST in relation to the origin of the sample locations and professions is shown in Table 2. It was also observed that the MRSE clone carriage was particularly high among nursing technicians, hemotherapy technicians and lab technicians, and MRSA clones were high only among hemotherapy technicians. All the MRSA and MRSE samples harboured the mec*A* gene.

**Table 2:** MRSA and MRSE ST distribution in relation to the sample location

Profession	n	Sample location/clones (ST)					
		Hands – ST type		Nasopharynx – ST type		Lab coat – ST type	
		MRSA	MRSE	MRSA	MRSE	MRSA	MRSE
Physician	2	**–**	59	**–**	17	**–**	–
Biochemist	1	**–**	–	**–**	644*	**–**	–
Nurse	2	**–**	642	**–**	663*	ST4952	–
Nursing technician	12	**–**	59**,** 653*	**–**	20, 173, 645*, 661*, 662*	**–**	20, 69, 152, 255, 646*
Nursing assistant	1	**–**	–	**–**	173	**–**	–
Hemotherapy technician	6	ST15	666*	**–**	17, 194, 668*	ST15, ST5800*^♦^ ST5 (*n* = 2) ^●^	17, 665*
Administrative staff	4	ST30	651*	**–**	647*, 664,20	**–**	–
Social worker	3	**–**	–	**–**	2, 61, 296	**–**	–
Biomedic	1	ST1993	670*	**–**	–	**–**	–
Lab technician	5	**–**	**–**	**–**	20, 643*, 648*, 652*, 185	ST5	–
Psychologist	1	**–**	–	**–**	667*	**–**	–
Biologist	1	**–**	–	**–**	671*	**–**	–
Cleaning staff	3	**–**	–	**–**	669*, 649*	**–**	650*
Ambulance driver	1	**–**	–	**–**	–	**–**	153
Physiotherapist	1	**–**	–	**–**	20	**–**	
Statistician	1	**–**	–	**–**	–	–	20

(−) Absence; (*) New sequence type (ST); (♦) New allele *pta* 680; (^●^) Corresponds to two STs in the same place

### Phylogenetic analysis

**Fig. 1:**
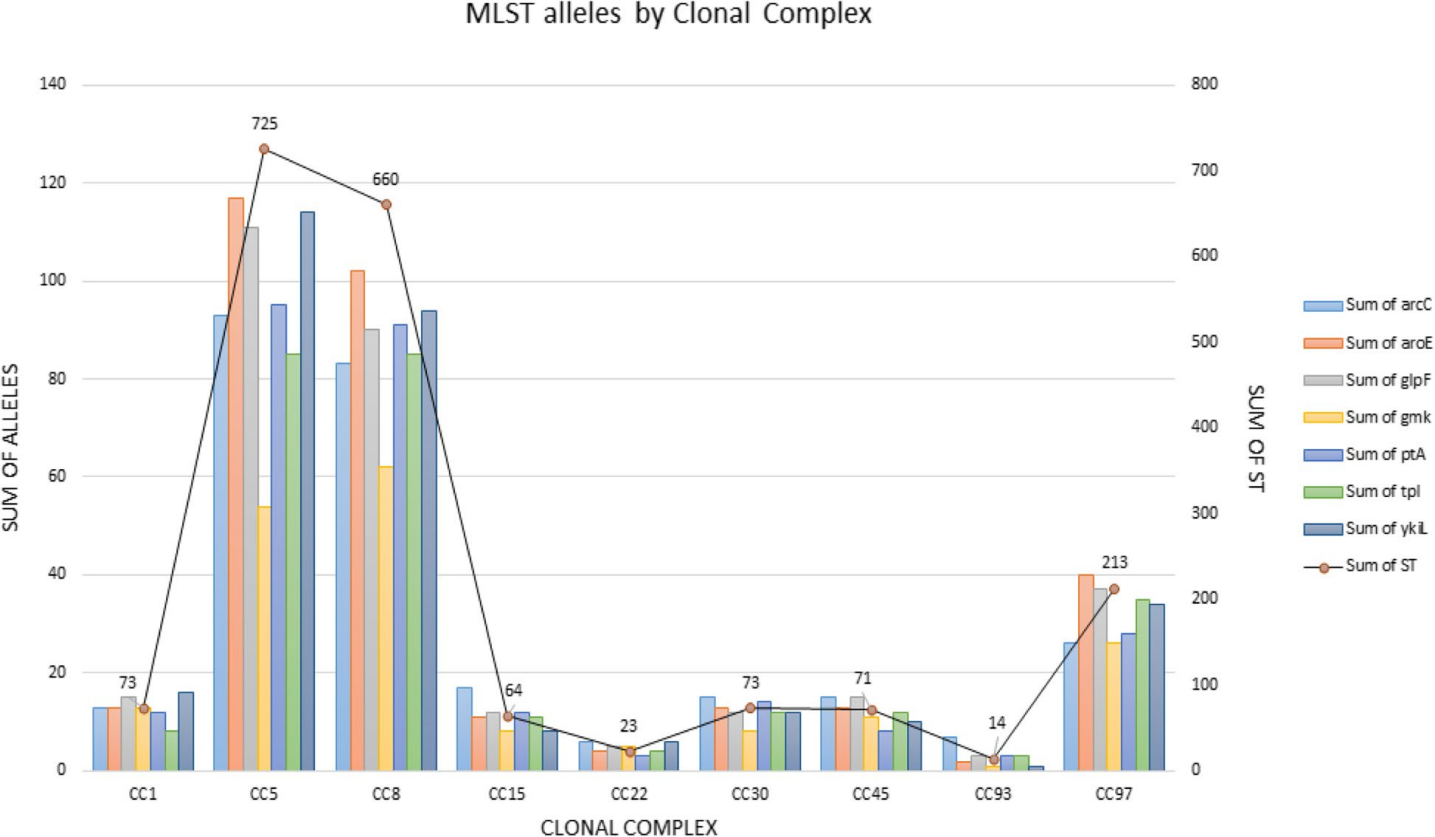
Distribution of ST and alleles of the seven MLST genes among clonal complexes of *S. aureus*

We observed that when comparing the sum of alleles and types of ST of each clonal complex from the 28,203 MRSA data bank sequences selected for this study, that the clonal complexes (CC) CC5 and CC8 presented the highest number of ST and presented most of the alleles of each MLST gene, as shown in Fig. 1. On the other hand, CC93 presented the lowest values of ST and alleles. The data revealed a strong positive correlation between the amount of STs and alleles of each analysed gene (R = 0.998241).

The Tajima neutrality test (Table 3), with significance *P* < 0.01, indicated that all the MLST regions are undergoing selection that favors an increase in the frequency of the most common allele and a decrease in the frequency of rare alleles for both *S. aureus* and *S. epidermidis*.

**Table 3:** Phylogenetic relationships between *S. aureus* clonal complexes, using the concatenated gene sequences from the MLST database

Microrganism	Gene	Nucleotide composition (%)				Lenght (n)	Variable sites (S)	S/n (Ps)	Θ	π	Tajima’s D
		A	C	G	T						
*S. aureus*	arcC	36.4	17.1	21.4	25.2	456	67	0.1469	0,0186	0.0068	−1.6654
	aroE	36.0	11.3	18.9	33.8	456	70	0.1535	0.0205	0.0099	−1.3612
	glpF	25.7	16.3	24.5	33.5	465	112	0.24	0.0318	0.0044	−2.3522
	gmK	37.2	12.4	21.2	29.3	417	53	0.127	0.0164	0.0083	−1.2761
	ptA	35.8	16.6	19.5	28.0	474	196	0.4135	0.0618	0.0068	−2.4876
	tpI	36.6	15.1	22.6	25.8	402	139	0.3458	0.0487	0.0096	−2.2167
	yqiL	34.0	15.6	22.4	28.0	516	68	0.1318	0.0162	0.0065	−1.5817
*S. epidermidis*	arcC	36.8	19.2	17.0	27.1	468	72	0.1538	0,0190	0.0153	−0.5227
	aroE	37.1	11.8	17.8	33.3	420	78	0.1857	0.0230	0.0110	−1.3911
	gtr	28.7	20.4	14.9	36.0	438	86	0.1963	0.0244	0.0119	−1.3708
	mutS	30.4	19.8	11.4	38.4	412	48	0.1165	0.0144	0.0054	−1.6011
	pyrR	34.4	16.1	21.9	27.5	424	57	0.1344	0.0167	0.0086	−1.2572
	tpiA	34.6	16.0	22.0	27.4	424	67	0.1580	0.0196	0.0036	−2.1526
	yqiL	31.6	15.2	21.5	31.6	416	62	0.1490	0.0185	0.0079	−1.4948

Nucleotide frequencies and parameters associated with the Tajima neutrality test for each MLST gene analysed. A, C, G and T are the percentages for each nucleotide in the genes in question. Size (n) corresponds to the total number of sites in each gene. Variable sites (S) correspond to the sites that showed nucleotide changes during alignments. Ps corresponds to the ratio S/n. Θ and π correspond to values that measure nucleotide diversity. Tajima’s D corresponds to the reference parameter to verify the neutral theory of evolution for each gene. All values of Tajima’s D showed statistical significance *P* < 0.01

**Fig. 2:**
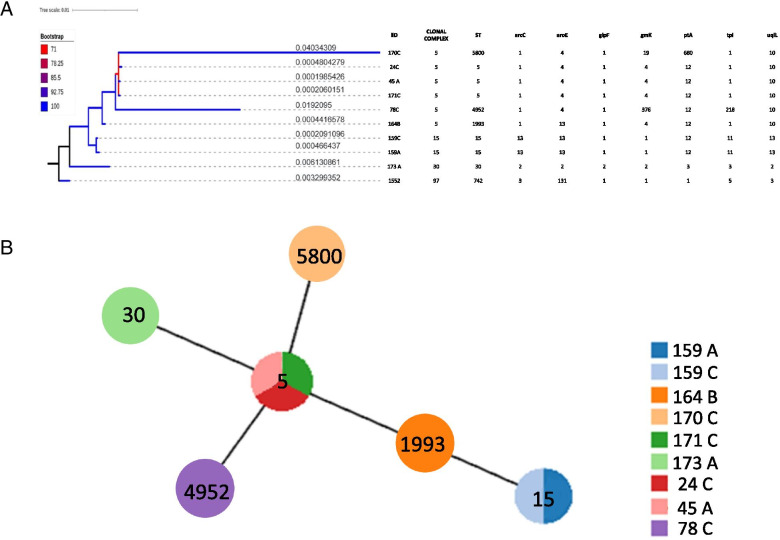
**A**. Phylogenetic tree obtained using Bayesian analysis with distribution of the MLST alleles for *S. aureus*. **B**. Distribution of ST using the e-Burst method

**Fig. 3:**
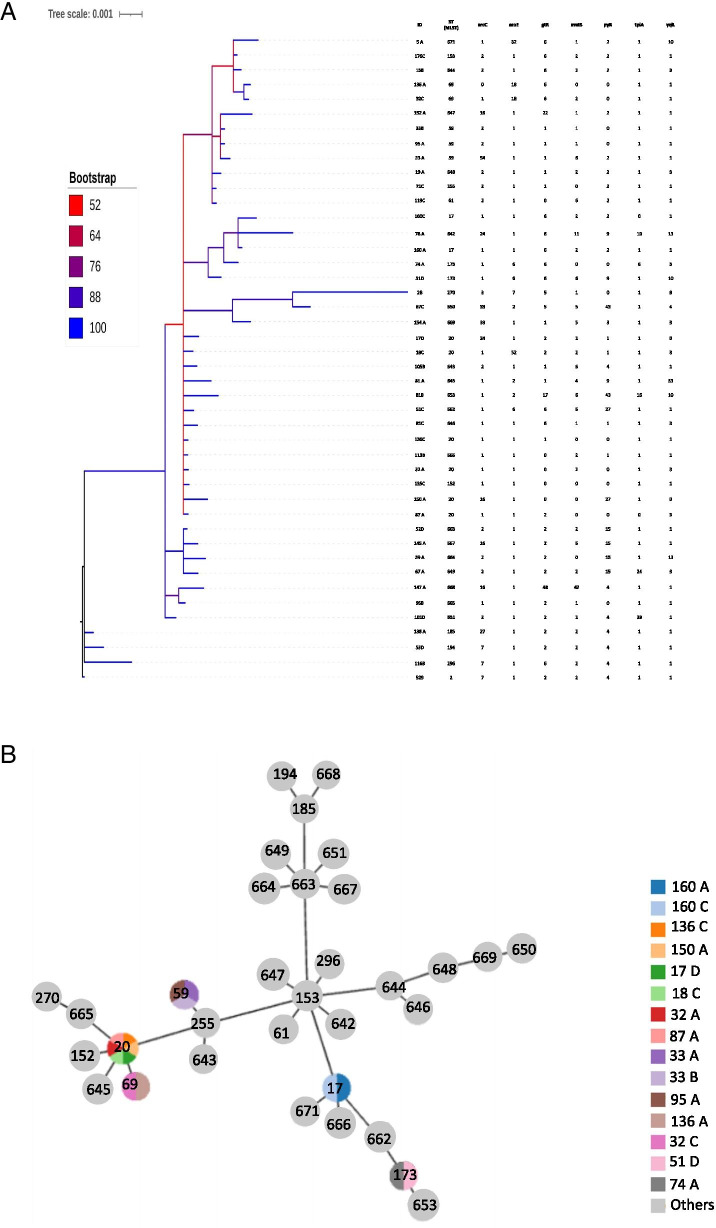
**A**. Phylogenetic tree obtained by Bayesian analysis with distribution of the MLST alleles for *S. epidermidis*. **B**. Distribution of ST using the e-Burst method

The allelic profile of each ST and CC is shown in Figs. 2 and 3, as well as the genetic proximity according to the phylogenetic tree. *S. aureus* STs were distributed in three clonal complexes: CC5 that included ST5, ST1993, ST4952 and ST5800; CC15 that included ST15 and CC30 that included only ST30. ST 742 (CC97) was used as an external group for the *S. aureus* phylogenetic analysis.

The phylogenetic tree (Fig. 2A) presents a topology that groups the sequences by CC, and reveals the monophyletic groups for the CC tree identified from *S. aureus*. The analysis of the e-Burst reveals the relationship among the STs, with ST5 as the founder of the others (Fig. 2B).

All of the *S. aureus* isolates that belong to CC5 (*n* = 6) showed 50% resistance to tetracycline, 66.6% to erythromycin, 33.3% to aztreonam and 83.3% to linezolid. The CC15 (*n* = 2) isolates showed resistance to tetracycline and aztreonam. The CC30 (*n* = 1) isolate was resistant to tetracycline, and showed sensibility to erythromycin, aztreonam and linezolid. None of the CC5, CC15 and CC30 isolates were susceptible to oxacillin, cefoxitin, penicillin and ampicillin.

The phylogenetic tree of *S. epidermidis* analysis group (ST2 as the external group - Fig. 3A) revealed the genetic proximity among the samples of *S. epidermidis* analysed in this study. We also observed strong monophyletic clusters formed between STs 17, 173 and 642; between STs 59 and 647 and also between STs 270, 650 and 669, which presented phylogenetic distance from the other sequences.

The e-Burst analysis revealed the relationship between the STs, with ST153 as the founder of the other STs (Fig. 3B). This analysis also showed that the samples of *S. epidermidis* were distributed in 33 different sequence types. STs 17, 20, 59, 69 and 173 presented the highest frequencies, with 2, 6, 3, 2 and 2 samples respectively (3B).

All of the 45 *S. epidermidis* STs showed resistance to oxacillin, cefoxitin; 97.7% of them were resistant to penicillin, 62.3% to erythromycin, 37.8% to tetracycline, and 35.5% to sulfazotrim. Resistance to vancomycin was not detected in any of the isolates.

## Discussion

MRSA is recognized as an important pathogen related to healthcare-associated infections. In fact, *S. epidermidis* has distinguished itself as an important pathogen and the most prevalent that has been isolated from the hospital infection process. This is due to the use of implanted devices, the increased number of immunocompromised patients [6, 22], and also because of the large proportion of nosocomial isolates with decreased susceptibility to glycopeptides or resistance to antibiotics [22, 23].

*S. epidermidis* tends to accumulate different antibiotic-resistance determinants, such as penicillin, clindamycin, macrolide, tetracycline, methicillin, mupirocin, and vancomycin [14]. Therefore, it is currently recognized as a reservoir that is capable of transferring resistant genes to *S. aureus* [24], which makes antibiotic therapy even more difficult, and often causes treatment failures [25, 26].

The staff at HEMOAM, as in other studies [6, 8], regardless of their work category, also harbour MRSE and MRSA isolates on their hands, lab coats, and in their nasopharynx. Regardless of genetic characteristics, the same clonal type of MRSA (ST5, ST15) was also detected at the same sample location (nasopharynx and lab coat). These findings are in agreement with other studies carried out in countries such as Sweden [27], Jordan, Egypt, Pakistan [28], Brazil [8] and China [29].

The presence of MRSE and MRSA on the hands facilitates the nasal colonization of these professionals, due to the common habit of introducing the fingers in the nose [11, 15, 28]. Contamination of the hands usually occurs at the workplace itself, during the execution of laboratorial procedures, direct contact with the patients as well as the environmental surfaces surrounding the hospitalized patients, through gloves, computer keyboards and telephones [11]. Due to this constant exposure, they are easily colonized on the skin or in the nose, which makes them important transmission vectors and sources of hospital outbreaks [10, 29], and this constant exposure puts them and their families at risk [8, 10].

Another transmission vector of MRSA and/or MRSE is the lab coat, used by clinicians, nurses and technicians who work at the hospitals as well as healthcare workers at HEMOAM. According to Singh et al. [30], patient-to-patient infections within hospitals and the community has been associated with the lodging of pathogens in lab coats of healthcare workers that also hang their coats in their cars, offices, or in improper environments outside the hospital areas such as in dining rooms, coffee shops and even shopping malls [31]. The seriousness of this problem is related to the fact that the majority of these professionals are not concerned with the risk of contamination either due to arrogance or ignorance of the basic concepts of microbiology [31].

Different studies [31, 32] have also demonstrated that lab coats become progressively contamined during their use, and that the survival of the pathogens can last 10 to 100 days, thus confirming the environmental persistence and genetic adaptation of these pathogenic isolates, thus favouring the emergence of successful epidemic strains.

Since this study was carried out at a blood bank, where hematological patients are treated, and whose treatment may compromise the immune system, even if only temporarily, it is important to note that both the MRSA and MRSE harboured the *mec*A gene. These molecular aspects have been described previously in the studies developed by Lee et al., Grace et al. and Ehlers et al. [5, 31, 33].

In this study, the prevalence levels of MRSA and MRSE were 3.16 and 100%, respectively. This is an important observation because, when compared with other studies, different results have been described. Among them, the studies carried out by Saffari et al.; Saadatian-Elahi et al.; Widerström et al.; Melendez et al.; Wang et al.; and Salman et al.; who identified levels of 70% MRSE; 5.3% MRSA; 5.3% MRSE, 85.5% MRSE, 30% MRSE and 9.3% MRSA, respectively [11, 12, 25, 26, 28, 34]. Certainly, this is a matter for concern, and one that requires strategic measures that can reduce the risks of transmission of these species in the blood bank.

In relation to MRSA, the high level of distribution occurred only among hemotherapy technicians (11.3%). This frequency is similar to that described by other authors, such as Khatri S. et al., who found MRSA levels of 10.5% in lab technicians and 9.9% in nurses, while El-Aila et al. found 30.4% in nurses and 16% in doctors. However, Salman et al. identified MRSA levels of 25% in laboratory staff and 10.8% in nurses, in Pakistan [28].

At the time of the collection of samples, the HEMOAM healthcare workers were harbouring different and resistant STs that could probably be spread in the hospital. Furthermore, as they work in different hospital units, control measures to avoid transmission of these kinds of bacteria is extremely necessary, but control can be difficult [35, 36] and, if due care is not taken with prevention protocols, the transmission of these pathogens to high-risk patients will cause the emergence of hospital outbreaks.

In relation to MRSE, the susceptibility tests showed high percentages of resistance to different antibiotics such as penicillin, tetracycline, sulfamethoxazole-trimethoprim, and erythromycin. Ehlers et al., Xu et al., and Guo et al. also reported similar levels of resistance when testing clinical isolates of MRSE [29, 37, 38].

AST for MRSA isolates showed resistance to ampicillin, tetracycline, erythromycin, penicillin, oxacillin and cefoxitin. Salman et al. reported a resistance profile to azithromycin, ceftriaxone, oxacillin, cefoxitin, co-amoxiclav and ciprofloxacin, while Monteiro et al. reported resistance to oxacillin, clindamycin, erythromycin, ciprofloxacin and gentamicin [28, 39].

Regarding molecular analysis, this research identified 23 new STs of MRSE (ST) in healthcare workers. Despite the genetic evolution, different epidemic clonal lineages of MRSE have been identified in different hospitals throughout the world, most of them belonging to the clonal complex (CC) 2, which were mainly composed of ST2. This is possibly due to genetic evolutions and adaptations of these bacteria to the hospital environment, the antibiotic therapy, hospitalization of the patient, migrations and hygienic conditions [12, 39–41].

It was observed that ST59 is the most frequent ST clone that dominates the hospital environment and the major clone responsible for nosocomial infections that have already been reported in other studies conducted in Hungary, Spain, Mexico, Greece Sweden, Africa, China [26, 33, 40–42].

The new STs ST644, ST645, ST648, ST666, ST661, ST671 and ST670 came from the common ancestor ST153, which has also already been identified in five isolates of *S. epidermidis* in Ireland, and harbours ACME IV [43]. ST699 was reported in several countries such as India, Russia, Ireland, Denmark and the United States, and ST649, ST664, ST651, ST668 also have been described in countries like Germany, Switzerland, Poland, India, and Brazil, thus confirming the worldwide dissemination of these clones [http://www.mlst.net].

In this study, CC5 (ST5 (*n* = 3), ST1993, ST5800**,** ST4952 (6/9) 66.6%) was the most common CC among MRSA isolates, with ST5 as the dominant clone. Gu et al. [29] in Shanghai also found 91.9% of CC5 MRSA isolates among patients with MRSA blood stream infections, while, in a clinical samples at Galway University hospital, Harrinson et al. [44] found ST5 isolates belonging to the CC5 MRSA.

In Spain, an analysis conducted by Montarelo et al. [45] over 15 years revealed that CC5 was also the most prevalent CC among MRSA isolates associated with methicillin resistance. In Latin American countries, with the exception of Ecuador, Brazil, and Colombia, CC5 MRSA was also prevalent in bloodstream infections [39, 40, 46, 47].

Recent studies have confirmed that multi-drug resistant and biofilm-producing pathogenic strains, which colonize inpatients or healthcare workers, caused different infectious processes in hospitals [38], and conventional antibiotics are not effective against biofilm, thus limiting therapeutic treatment [28]. Therefore, studies of the molecular epidemiology of pathogenic clones present in the hospital environment is of extreme importance, since the colonization of patients and healthcare workers may contribute to the emergence of nosocomial infections.

This study is important because, even with the measures taken to prevent hospital infection, it has shown the presence of pathogenic bacteria, such as MRSE and MRSA, in healthcare workers at HEMOAM, and the results will certainly contribute to improvements in the standard control measure protocols in order to avoid possible transmission to patients.

Currently, the hospital infection control committee (CCIH) at HEMOAM monitors multi-resistant bacteria and the relevant preventive measures. Molecular protocols for the identification of multidrug-resistant pathogens are also being implemented to avoid infective treatment.

## Conclusion

Our data desmonstrated the presence of pathogenic clones of MRSE and MRSA in healthcare workers at HEMOAM. It also showed that these professionals were a possible source of transmission and dissemination of these clones at the blood bank. Even with the control measures currently in use, the need for awareness and improvement of them is particularly important to prevent the colonization of these professionals and the likely transmittion to their families, colleagues and patients. This study also demonstrated the importance of identifying pathogenic clones of bacteria, their antimicrobial profile, and the use of the molecular technique, which can improve hospital care by preventing transmission and possible outbreaks.

## Supplementary Information

**Additional file 1: Table S1.** Multivariate logistic regression model for MRSE.

## Data Availability

The dataset(s) supporting the conclusions of this article is (are) included within the article (and its additional file(s)). GenBank accession numbers: MW115983–115989; 115990–115996; 115997–116003; 116004–116010; 116011–116017; 116018–116024; 116025–116031; 116032–116038; 116039–116045. https://www.ncbi.nlm.nih.gov.

## Abbreviations

ABI 3130: Genetic Analyser 3130 Applied Biosystems
AST: Antimicrobial susceptibility test
ATCC: American Type Culture Collection
A, T, C, G: Adenine, cytosine, thymine and guanine.
BHI: Brain Heart Infusion
CC: Clonal complexes
CCIH: Hospital infection control committee
CDC: Center for Disease Control and Prevention
CLSI: Clinical and Laboratory Standards Institute
DNA: Deoxyribonucleic acid
eBURST: An algorithm that predicts the founding (ancestral) genotype.
GTR: General Time Reversible
HCAI: Health care-associated infections
HEMOAM: Hematology and Hemotherapy Foundation of the Amazonas
ICU: Intensive care unit
MAFFT: Multiple alignment software program for amino acid or nucleotide sequences
mecA: Part of staphylococcal chromosome cassette mec (SCCmec)
MIC: Minimum inhibitory concentration
MLST: Multilocus sequence typing
MRSA: Methicillin-resistant *S. aureus*s
MRSE: Methicillin-resistant *S. epidermidis*
ST: Sequence Type
VITEK-2: Compact automated system for antimicrobial identification and susceptibility tests
USA: United States of America
