# Supplementary material for: Molecular epidemiology of methicillin resistant Staphylococcus species in healthcare workers of a blood bank in the Brazilian Amazon
Source: BMC Microbiol. 2021 Nov 4;21:306. doi: 10.1186/s12866-021-02365-1 (PMC8567558; doi:10.1186/s12866-021-02365-1)
Supplement: Supplementary file 1 — Additional file 1: Table S1. Multivariate logistic regression model for MRSE. [file 12866_2021_2365_MOESM1_ESM.docx]

| Table S1. Multivariate logistic regression model for MRSE | | | | |
| --- | --- | --- | --- | --- |
| MRSE | aOR | 95% CI | | *P* |
| Male | 0.328 | 0.057 | 1.873 | 0.210 |
| Age | 1.001 | 0.914 | 1.095 | 0.989 |
| Function |  |  |  |  |
| Support | 1 | - | - | - |
| Clinical | 3.071 | 0.154 | 61.179 | 0.462 |
| Laboratory | 0.418 | 0.034 | 5.122 | 0.495 |

aOR: Adjusted Odds Ratio; Function: Professions were allocated by function as administrative,support clinical, and laboratory staff
